# Posterior petrosectomy for resection of pontine cavernous malformation

**DOI:** 10.3171/2019.10.FocusVid.19389

**Published:** 2019-10-01

**Authors:** Avital Perry, Thomas J. Sorenson, Christopher S. Graffeo, Colin L. Driscoll, Michael J. Link

**Affiliations:** 1 Department of Neurologic Surgery, Mayo Clinic, Rochester;; 2 School of Medicine, University of Minnesota, Minneapolis; and; 3 Department of Otorhinolaryngology, Mayo Clinic, Rochester, Minnesota

**Keywords:** hemorrhage, cavernous malformation, brainstem, posterior petrosectomy, microsurgery, video

## Abstract

Cavernous malformations (CMs) are low-pressure, focal, vascular lesions that may occur within the brainstem and require treatment, which can be a substantial challenge. Herein, we demonstrate the surgical resection of a hemorrhaged brainstem CM through a posterior petrosectomy approach. After dissection of the overlying vascular and meningeal structures, a safe entry zone into the brainstem is identified based on local anatomy and intraoperative neuronavigation. Small ultrasound probes can also be useful for obtaining real-time intraoperative feedback. The CM is internally debulked and resected in a piecemeal fashion through an opening smaller than the CM itself. As brainstem CMs are challenging lesions, knowledge of several surgical nuances and adoption of careful microsurgical techniques are requisite for success.

The video can be found here: https://youtu.be/szB6YpzkuCo.

## Transcript

This case demonstrates a posterior petrosal approach for a large brainstem cavernous malformation **(0:27)**. The patient is a 20-year-old young man with pertinent history of a left temporal fibrillary astrocytoma treated 8 years prior to presentation. He had undergone a near-total resection of his tumor followed by 5940 cGy of fractionated radiotherapy. He presented with dramatic new neurological deficits of right facial weakness, right upper extremity dysmetria and weakness, and severe headache **(1:00)**. Imaging included CT and an MRI scan, which showed a large hemorrhage almost completely replacing the pons. It was centered more toward the patient’s left **(1:17)**. A left temporal occipital craniotomy and retrolabrynthine presigmoid exposure was performed. The dura was opened along the inferior temporal lobe, which was very adherent to the tentorium from the patient’s prior surgery and radiation. The presigmoid dura was also opened and then CSF was released from the cisterns to improve intracranial relaxation **(1:46)**. CN VIII can be seen in the center of the exposure and the arachnoid above this widely opened. The superior petrosal vein and sinus is doubly ligated with clips and then the tentorium is incised all the way to the tentorial incisura, taking great care not to damage CN IV. This allows a much wider avenue into the posterior fossa **(3:12)**. The arachnoid around the petrosal vein is then opened to isolate this vessel. The petrosal vein is blocking our entry into brainstem and is therefore coagulated and divided. A large loop of the anterior inferior cerebellar artery is also overlying the desired entry zone into the lateral brainstem, so this is also mobilized up off the brainstem to be able to have a good safe entry zone. The petrosal vein has to be coagulated and divided more proximal to free up the anterior inferior cerebellar artery without damage. Having divided the tentorium, this allows the sagittal sinus to be retracted posteriorly, which allows a wider working avenue into the posterior fossa. The arachnoid around CN VIII down toward the jugular foramen is widely opened, which takes traction off CN VIII, and, since this is hearing preservation operation, this is preferable. We now have wide look at the brainstem and the pertinent vasculature can be mobilized up off the brainstem and moved inferiorly **(3:44)**. Frameless stereotaxis confirms the trajectory. The location of the myelotomy is marked with bipolar, and the lateral brainstem is opened with scissors between CNs V and VIII. The opening in the brainstem is kept purposefully small as it always seems to get larger as we work **(4:11)**. After just a couple millimeters, we encounter fresh blood clot and the lesion. Much of it can be easily removed with gentle suction and irrigation. Using gentle dissection, the walls of the malformation can be established against the normal white matter of the brainstem. Most of these lesions are removed in a piecemeal fashion **(4:49)**. Bipolar coagulation can be helpful to shrink the lesion down or to eliminate some of the very small feeding or draining vessels going into the lesion. As the periphery of the lesion is identified, it can be removed with just gentle traction and a cup punch **(5:27)**. If the lesion does not come easily, it is better to halt and use microscissors to divide the lesion particularly when removing from brainstem. Once this is done, however, it usually comes out in fairly large pieces, which ensures complete resection **(6:11)**. The cavity needs to be carefully inspected to make sure that no cavernoma is left behind. Once this is performed, then the resection cavity is lined with Surgicel and a thin layer of fibrin sealant is placed in this to aid in hemostasis **(6:47)**. Postoperative MRI shows complete removal of lesion. The brainstem resumes much more natural, normal configuration once the lesion is removed **(7:02)**. The patient did very well after this procedure. With near-complete immediate improvement in neurological deficits. He remained neurologically stable with no recurrent bleeding now 3 years following operation.
